# Kidney Transplants From Donors on Extracorporeal Membrane Oxygenation Prior to Death Are Associated With Better Long-Term Renal Function Compared to Donors After Circulatory Death

**DOI:** 10.3389/ti.2021.10179

**Published:** 2022-02-08

**Authors:** Marilena Gregorini, Elena Ticozzelli, Massimo Abelli, Maria A. Grignano, Eleonora F. Pattonieri, Alessandro Giacomoni, Luciano De Carlis, Antonio Dell’Acqua, Rossana Caldara, Carlo Socci, Andrea Bottazzi, Carmelo Libetta, Vincenzo Sepe, Stefano Malabarba, Federica Manzoni, Catherine Klersy, Giuseppe Piccolo, Teresa Rampino

**Affiliations:** ^1^ Dipartimento di Medicina Interna e Terapia Medica, Università Degli Studi di Pavia, Pavia, Italy; ^2^ Unit of Nephrology, Dialysis and Transplant, San Matteo Hospital Foundation (IRCCS), Pavia, Italy; ^3^ Unit of General Surgery 2, Department of Surgical Sciences, San Matteo Hospital Foundation (IRCCS), Pavia, Italy; ^4^ Transplant Unit, IRCCS Fondazione Policlinico San Matteo, Pavia, Italy; ^5^ Transplant Center, Department of General Surgery and Abdominal Transplantation, Niguarda Cà Granda Hospital, Milan, Italy; ^6^ Department of Medicine and Surgery, University of Milano Bicocca, Milan, Italy; ^7^ Department of Anesthesia and Critical Care, San Raffaele Scientific Institute (IRCCS), Milan, Italy; ^8^ Transplant Unit, Department of General Medicine, San Raffaele Scientific Institute, Vita‐Salute San Raffaele University, Milan, Italy; ^9^ Department of Surgery, San Raffaele Scientific Institute, Vita‐Salute San Raffaele University, Milan, Italy; ^10^ ICU1 Department of Intensive Medicine, Foundation IRCCS Policlinico San Matteo, Pavia, Italy; ^11^ Health Promotion, Environmental Epidemiology Unit, Hygiene and Health Prevention Department, Health Protection Agency, Pavia, Italy; ^12^ Biometry and Clinical Epidemiology Service, Foundation IRCCS Policlinico San Matteo, Pavia, Italy; ^13^ Regional Transplant Coordination, Milan, Italy

**Keywords:** donation after circulatory death, extracorporeal membrane oxygenation, renal transplantation, hypothermic perfusion, eGFR

## Abstract

Donation after circulatory death (DCD) allows expansion of the donor pool. We report on 11 years of Italian experience by comparing the outcome of grafts from DCD and extracorporeal membrane oxygenation (ECMO) prior to death donation (EPD), a new donor category. We studied 58 kidney recipients from DCD or EPD and collected donor/recipient clinical characteristics. Primary non function (PNF) and delayed graft function (DGF) rates, dialysis need, hospitalization duration, and patient and graft survival rates were compared. The estimated glomerular filtration rate (eGFR) was measured throughout the follow-up. Better clinical outcomes were achieved with EPD than with DCD despite similar graft and patient survival rates The total warm ischemia time (WIT) was longer in the DCD group than in the EPD group. Pure WIT was the highest in the class II group. The DGF rate was higher in the DCD group than in the EPD group. PNF rate was similar in the groups. Dialysis need was the greatest and hospitalization the longest in the class II DCD group. eGFR was lower in the class II DCD group than in the EPD group. Our results indicate good clinical outcomes of kidney transplants from DCD despite the long “no-touch period” and show that ECMO in the procurement phase improves graft outcome, suggesting EPD as a source for pool expansion.

## Graphical Abstract

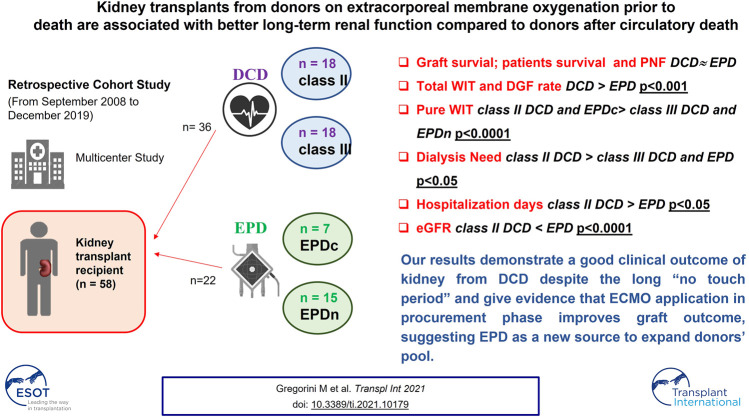

## Introduction

Organ shortage remains the main obstacle in kidney transplantation, thus there is an urgent need for donor pool expansion. Donation after circulatory death (DCD) serves as an additional organ source and has become the current medical practice, despite each country having its own DCD protocol according to its own legislation and healthcare facilities (1). A major difference in the DCD protocols of countries is the “no-touch” period duration, i.e., the time required by law for the circulatory death declaration. Although ethical and practical issues assume the maximal relevance in this type of donation setting, the “no-touch” time ranges from 5 to 30 min in Russia. In Italy, it is 20 min, which is the second longest interval (2, 3). DCD has not been considered in Italy for many years, because of such a prolonged warm ischemia time (WIT); the argument being that it was too long for organ survival. A prolonged WIT is associated with a high rate of organ discard, primary non function (PNF), and delayed graft function (DGF) of kidney transplants from DCD, even if graft and recipient survival is comparable to that linked to donation after brain death (DBD) (4). Due to these complexities, we sought to determine a particular type of donor from among the existing Maastricht categories. It would typically be a patient in whom an advanced resuscitation attempt through extracorporeal life support (ECLS) using extracorporeal membrane oxygenation (ECMO) has failed.

ECLS is an advanced resuscitation technique that ensures blood circulation in asystolic patients. It is a total-body cardiopulmonary by-pass system, applied to allow brain perfusion while cardiac activity restoration is attempted in case of severe heart or lung failure (5). If resuscitation is unsuccessful, these patients become donors with a total-body ECMO already activated. On obtaining family consent, total-body ECMO is switched with normothermic regional perfusion (NRP) by inserting an aortic balloon above the celiac trunk to maintain only abdominal perfusion. These donors do not fit any existing Maastricht criterion because artificial blood circulation starts from cardiac arrest until the patient’s death. These patients are always hospitalized in the intensive care unit (ICU) and can be on ECMO for several hours to weeks. Therefore, they are named “donors on ECMO prior to death” (EPD). In this setting, according to Italian legislation, death can be declared by applying cardiac or neurological (EPDc or EPDn, respectively) criteria. When using the latter criteria there is no need to stop circulation while recording the electrocardiogram (EKG) for 20 min, therefore warm ischemia time is shorter.

To our knowledge, there are no studies on EPD as a new potential donor category. Here we report our pragmatic experience over 11 years, comparing graft outcomes achieved with EPD to those achieved with DCD.

## Patients and Methods

A total of 58 patients transplanted with kidneys harvested from DCD (36) and donors on EPD (22) between January 2008 and December 2019 were studied. There were 31 donors from DCD (10 recipients received kidneys from five DCD donors), and 21 donors on EPD (2 recipients received kidneys from one donor on EPD). The transplants were performed in three different kidney transplant centers: Foundation IRCCS Policlinico San Matteo Hospital (Pavia), Niguarda Ca’ Granda Hospital (Milan), and San Raffaele Hospital (Milan). The study protocol was approved by the ethics committee (p-20200027199) and fully complied with the 2000 Declaration of Helsinki (6).

DCD categories were defined according to the Maastricht criteria (7). Donors on EPD were recruited from ICU patients, who had undergone an advanced resuscitation protocol, including total-body arterio-venous ECMO as part of ECLS to treat cardiac arrest or severe heart failure. When ECLS therapy failed, the patients became suitable donors.

In this situation, according to Italian legislation, death can be declared using circulatory or neurological criteria. Circulatory death is legally defined as an irreversible cessation of circulatory function, based on definitive proof obtained using an electrocardiogram (EKG). An observation period of 20 min (“no-touch” period), as Italian law imposes, was employed to ensure the irreversibility and permanence of patient’s circulatory death. In cases of donors on EPD, death certification was based on neurological criteria; brain death was declared according to international standardized methods (8). Once the consent for donation was obtained, after death declaration, an aortic balloon was inserted through the contralateral iliac artery to ensure selective abdominal circulation and ECMO was restarted to provide NRP (1–4 h) for *in situ* kidney preservation.

Then all kidneys were placed in a hypothermic perfusion machine (HPM RM3-Water Medical System IGL, Lissieu, France and W.A.V.E.S. Water Medical System IGL, Lissieu France) except for the first three procured at the Pavia center, which were preserved in static cold storage because HPM was not yet available.

To avoid PNF, the kidneys were evaluated per donor history, macroscopic appearance, histological criteria and, most importantly, perfusion parameters (9). In particular, the histological pre-transplant examination was performed by obtaining a wedge biopsy specimen from the renal superior pole scoring by using the Remuzzi classification (10). Vascular thrombosis and/or a Remuzzi score of >4 were criteria for organ discard.

The kidneys were also evaluated using perfusion parameters: systolic perfusion pressure was set at 35–40 mmHg. Renal resistance (RR) values were machine-calculated in real-time, as a ratio between the mean perfusion pressure (mmHg) and the flow through the kidney (ml/min); values were recorded every 60 s throughout the perfusion. The perfusion liquid used was a modified Beltzer solution (Perfgen Institut Georges Lopez, Lissieu, France). The perfusion temperature was set at 4–6°C. When the RR value fell below 0.4 mmHg/ml/min, the kidneys were considered suitable for transplantation. In contrast if the RR value remained high after 6 h of perfusion, the kidneys were considered unsuitable for transplantation and discarded, regardless of the biopsy result. The kidneys were detached from the HPM just a few minutes before starting vascular anastomoses.

**FIGURE 1:**
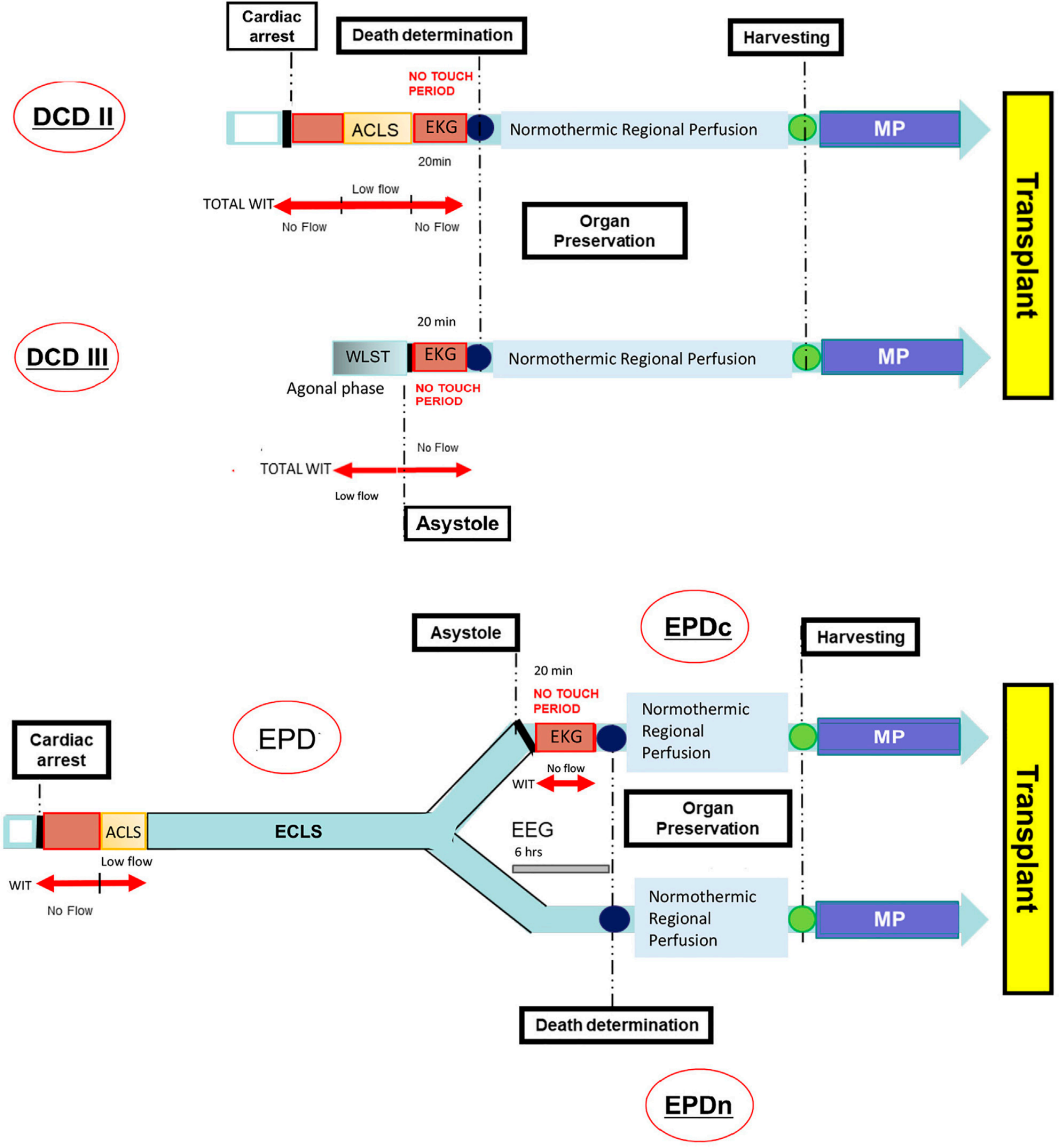
Timeline from the cardiac arrest to the organ transplant in DCD and EPD. ACLS, Advanced Cardiovascular Life Support; DCD, Donation after Circulatory Death; DCDII, Maastricht class II DCD; DCDIII, Maastricht class III DCD; ECLS, Extracorporeal Life Support; EEG, Electroencephalography; EKG, Electrocardiogram; EPD, Extracorporeal Membrane Oxygenation (ECMO) Prior to Death Donation/Donor; EPDc, Patient’s death certified by cardiac criteria; EPDn, Patient’s death certified by neurological criteria; MP, Machine Perfusion; WIT, Warm Ischemia Time; WLST, Withdrawal Life Sustaining Treatment.

Figure 1 shows the timeline and event order for the DCD and EPD transplant programs.

## Clinical Variables

### Donors

Donor-related variables included: age, body mass index (BMI), sex, death causes, comorbidities, and Maastricht criteria for DCD.

Donors on EPD were distinguished by EPDn and EPDc depending on the death certification.

### Recipients

The recipients were divided into: Maastricht class II DCD, class III DCD, and EPDc and EPDn, according to the donor type. Recipient-related variables included: age, BMI, sex, dialytic age (months), human leukocyte antigen (HLA) match, maximum panel reactive antibody, comorbidities and, primary renal disease.

### Transplant-Related Variables

- Total WIT: time elapsed between the moment of cardiac arrest and the beginning of organ preservation by NRP.

The total WIT of the EPD group did not include the total-body ECMO time.

-Pure WIT or no-flow period: asystolic period without any resuscitation maneuvers. This time comprised the first variable period of no flow, the duration of which depends on the celerity in beginning cardiopulmonary resuscitation and a second unchanged period which corresponds to a 20 min recording of EKG.
-The low-flow period: period in which organ blood perfusion was maintained using cardiopulmonary resuscitation, advanced cardiac life support, and chest compression in an attempt to save the patient’s life.
-The no-touch period: stand-off time without any intervention to certify the patient’s death per circulatory death criteria.
-Cold ischemia time (CIT): time from the beginning of organ perfusion, using cold preservation solutions during the organ retrieval surgery, until the end of graft perfusion through HPM.

Data related to these time intervals, RR, and flow parameters during hypothermic perfusion were collected.

### Kidney Suitability for Transplantation

Donor kidneys were discarded according to one of the following criteria:

(1) machine perfusion pump parameters: persistent flow rate of <60 ml/min and/or resistance index of>0.4 mmHg/ml/min;
(2) vascular thrombosis identified upon biopsy analysis performed before hypothermic perfusion; and
(3) Remuzzi score >4.

### Immunosuppressive Regimen

Anti-thymocyte globulin or basiliximab was administered as immunosuppression induction therapy. Additionally, methylprednisolone (500 mg/day) was administered intravenously on the first day; its dose was progressively decreased until day 6. Oral methylprednisolone (16 mg) administration was introduced on post-transplant day 7 and reduced every 3 months, to reach a maintenance dose of 4 mg/day. It was discontinued after 1 year (except in cases of previous acute rejection, re-transplantation, or glomerulonephritis as the primary renal disease). Mycophenolate mofetil was administered at a daily dose of 1–2 g. Tacrolimus or cyclosporine was started on day 1 at 0.1–0.15 mg/kg/day or 6–8 mg/kg/day, respectively. The dose was adjusted to achieve a therapeutic target blood trough level (tacrolimus 8–10 ng/ml, cyclosporine 150–200 ng/ml in the first 3 months). Maintenance immunosuppression regimens consisted of calcineurin and/or mTOR inhibitors, mycophenolate mofetil, and methylprednisolone. Immunosuppressor trough levels were reduced based on transplant age.

### Follow-Up

The kidney recipients were followed up for 1–11 years. HLA mismatch between donors and recipients was categorized according to differences at the HLA-A, HLA-B, HLA-DR, and HLA-DQ loci.

Routine blood tests and serum levels of immunosuppressive drugs were regularly assessed. Follow-up ceased due to patient death, PNF, or graft failure.

The following end points were evaluated after transplantation:

-PNF was defined as the immediate failure of the graft function, requiring permanent dialysis or a re-transplantation;
-DGF was defined as a need for dialysis within the first week after transplantation, DGF duration was measured as the number of dialysis sessions
-Dialysis need was defined as the number of dialysis sessions required after transplantation;
-Length of hospital stay in the post-transplant period was determined based on the number of days; and
-Graft survival rate was defined as the time from transplantation to graft nephrectomy, return to dialysis, or re-transplantation. It did not cover patient death with a functioning graft.

Incidences of PNF, DGF, and acute rejection were retrospectively analyzed in all groups of patients. Acute rejection biopsies were classified according to the Banff 2013 classification (11).

In addition, severe post-operative complications such as viral and bacterial infections, severe bleeding, renal vein/artery thrombosis, stenosis of the bladder-ureter anastomosis, allograft rupture, lymphorrhagia, and urine leakage were analyzed retrospectively.

### Assessment of Graft Function

Estimated glomerular filtration rate (eGFR), calculated using the Chronic Kidney Disease Epidemiology Collaboration (CKD-EPI) formula (expressed in milliliters per minute and adjusted for body surface area), was determined on days 7, 1, 3, 6, and 12 months after transplantation and every year throughout the follow up period.

A percutaneous renal graft biopsy was performed 2 or 3 weeks after transplantation when DGF persisted. Furthermore, a biopsy was performed for patients presenting with an abrupt decrease or a lengthy deterioration of renal function, significant proteinuria, or finally, the appearance of specific antibodies against the donor during the follow-up period.

### Statistical Analysis

Parametric variables are expressed as mean and standard deviation or standard error values and non-parametric variables as medians and interquartile ranges. The categorical variables are expressed as percentages. Chi-square or Fisher test was used for comparative analysis of categorical variables. Differences in eGFR were evaluated using repeated-measures ANOVA.

Patient and graft survival rates were estimated using the Kaplan-Meyer method, the differences were compared using the log rank test. All tests were two-tailed and considered statistically significant at *p* < 0.05.

## Results

From September 2008 to December 2019, 58 kidney transplants were performed; of which 36 kidneys came from DCD and 22 kidneys from EPD. According to the Maastricht DCD criteria, 18 donors each belonged to classes II and III. In the EPD group, death was certified by circulatory criteria in seven donors and by neurological criteria in 15 donors.

### Baseline Characteristics

As shown in Table 1, baseline characteristics were similar in the groups. All recipients were Caucasian. All donor deaths were caused by cardiac arrest and cardiogenic shock. Basiliximab and/or rabbit ATG were used for induction therapy (basiliximab: 69.4 and 68.8% of DCD and EPD recipients, respectively; rabbit ATG: 30.6 and 31.2% of DCD and EPD recipients, respectively; *p* = 0.7).

**TABLE 1:** Clinical and demographic baseline characteristics in DCD and EPD groups.

Variables	Class II DCD group (*n* = 18)	Class III DCD group (*n* = 18)	EPD group (*n* = 22)	*p* value
Donor age (years) (m ± SD)	49.6 ± 7.94	54.89 ± 8.7	48 ± 11.9	*p* = 0.08
Recipient age (years) (m ± SD)	55.89 ± 10.9	55.61 ± 8.7	50.86 ± 10.34	*p* = 0.25
Donor BMI (m ± SD)	27.05 ± 3.4	26.48 ± 3.65	26.51 ± 2.8	*p* = 0.85
Recipient BMI (m ± SD	25.02 ± 5.1	24.75 ± 3.58	23.33 ± 3.24	*p* = 0.37
Donor gender (%)				*p* = 0.7
Male	79.4	75.1	86.3	
Female	22.6	24.9	13.7	
Recipient gender (%)				*p* = 0.46
Male	66.6	62.5	68.1	
Female	33.4	37.5	31.9	
Donor comorbidity (%)				
Diabetes	5.5	7.3	9	*p* = 0.6
Hypertension	28.7	29.5	23.6	*p* = 0.32
Cardiovascular disease	27.5	23.5	31.8	*p* = 0.07
Dyslipidemia	16.6	17.7	15.6	*p* = 0.69
Current smoking	21.7	22	20	*p* = 0.1
Recipient comorbidity (%)				
Diabetes	5	6	3	*p* = 0.52
Hypertension	77.7	72.3	60.0	*p* = 0.11
Cardiovascular disease	22.2	18	19.9	*p* = 0.42
Dyslipidemia	8.3	10	10.1	*p* = 0.40
Current smoking	19.4	8	10	*p* = 0.50
Recipient dialytic age (months) (mean ± SD)	39 ± 5.3	37 ± 8	42.8 ± 5.8	*p* = 0.42
HLA D/R matches (median and IQR)	2 (1–3)	2 (1–3.7)	2 (1–2,25)	*p* = 0.7
Maximum panel reactive antibody (median; min-max)	0 (0–65)	0 (0–50)	0 (0–65)	*p* = 0.49
Primary renal disease (%)				
Polycystic kidney disease	19.4	24.5	30.3	*p* = 0.5
Glomerulonephritis	25.8	18.8	8.6	*p* = 0.13
Nephroangiosclerosis	29	29	22.2	*p* = 0.74
Unknown	6.5	10.5	27.8	*p* = 0.10
Miscellaneous	19.3	17.2	11.1	*p* = 0.12

### Donor Kidney-Related Variables

As shown in Table 2, total WIT was the longest in the class II DCD group and the shortest in the EPDn group (*p* < 0.0001).

**TABLE 2:** Transplant-related variables in DCD and EPD groups.

	DCDII	DCDIII	EPD	*p*-value
Total WIT (minutes, m ± SD)	142 ± 40°	60.5 ± 8.1*	25.25 ± 9.3	°*p* < 0.0001 vs. DCDIII, EPD
				*p < 0.001 vs. EPD
Pure WIT (minutes, m ± SD)	28 ± 3.1*	20.8 ± 3.1°	19.8 ± 7.13	*p < 0.0005 EPD
				°*p* < 0.0001 vs. DCDII
CIT (minutes, median, and IQR)	1,065 (540–1,440)	975 (660–1,440)	1,080 (915–1,230)	NS
TB ECMO time (hours, median, and IQR)		36 (19.88–63.38)		
NRP time (minutes, median, and IQR)	210 (190–230)	240 (220–250)	200 (180–230)	NS
HMP time (minutes, median, and IQR)	720 (330–1,260)	660 (375–1,380)	1,080 (915–1,230)	NS
Perfusion parameters	DCD	EPD		
Flow (ml/min, m ± SD)	96.13 ± 27.55	82.69 ± 21.26	NS	
RR (m ± SD mmHg/mlmin^−1^)	0.25 ± 0.09	0.28 ± 0.12	NS	

The symbols * and ° refer to the statistical significance levels reported in *p* value column. WIT, warm ischemia time; CIT, cold ischemia time, TB ECMO, total body extracorporeal membrane oxygenation; NRP, normothermic regional perfusion; HPM, hypothermic perfusion machine; RR, renal resistance; SD, standard deviation; IQR, interquartile range.

Similarly, pure WIT was the longest in the class II DCD group and the shortest in the EPDn group (*p* < 0.0001). TB ECMO time was similar in the two EPD subgroups (EPDc and EPDn).

The groups showed no significant differences in CIT, NRP time, and perfusion parameters (flow and RR).

### Clinical Outcomes

PNF occurred in two patients, one in the class II DCD group (2.7%) and the other in the EPDc group (4.5%). In both cases, renal biopsy revealed ischemic coagulation necrosis.

**FIGURE 2:**
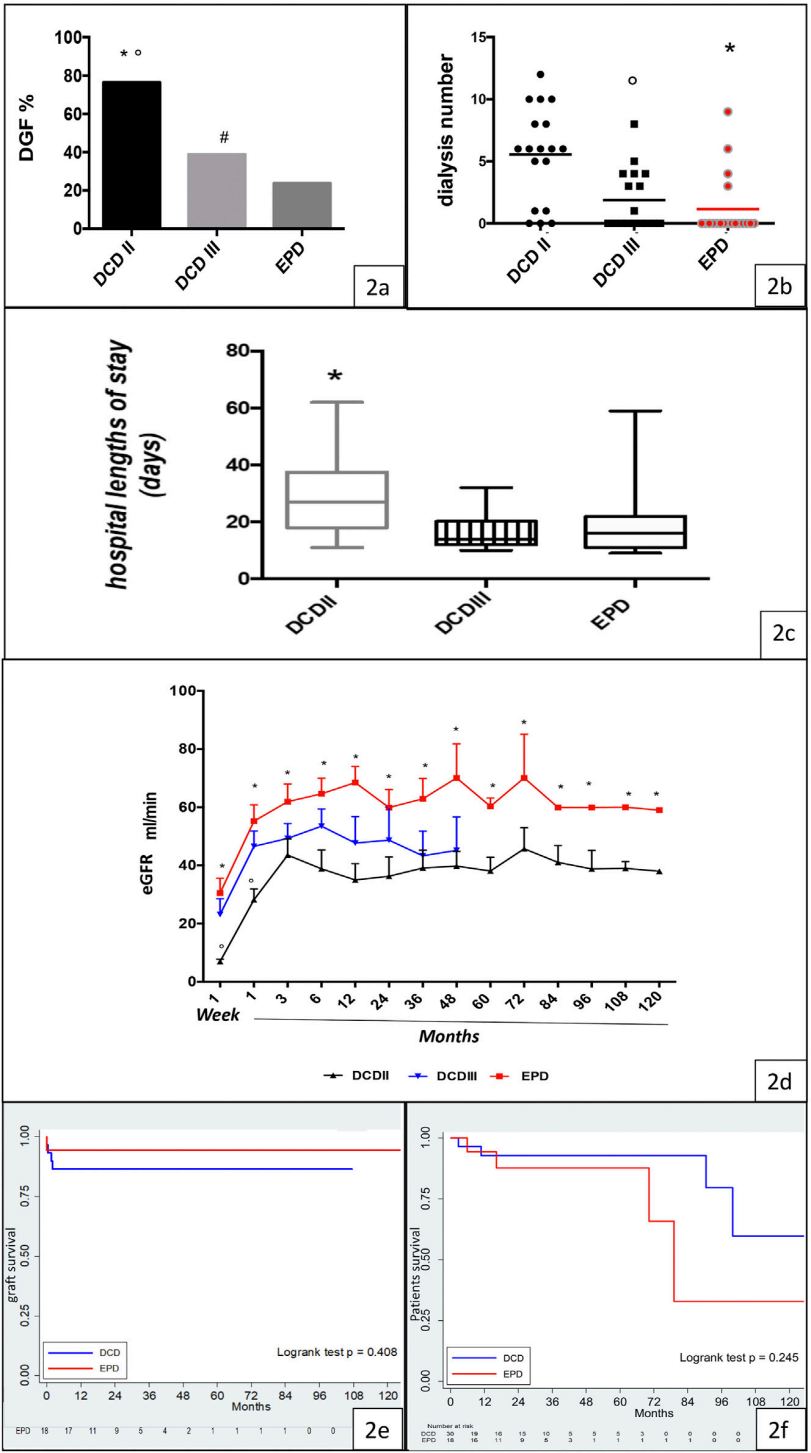
Clinical outcome, graft function, and graft and patient survival in the DCD and EPD groups. **(A)** DGF rate in the Maastricht class II DCD, Maastricht class III DCD, and EPD groups (DCDII vs. DCDIII, *p < 0.0001; DCDII vs. EPD, °*p* < 0.0001; DCDIII vs. EPD, #*p* < 0.0001). **(B)** Dialysis requirement in Maastricht class II DCD, Maastricht class III DCD, and EPD recipients (EPD vs. DCDII, *p < 0.001; DCDIII vs. DCDII, °*p* < 0.05). **(C)** Hospital length of stay in the studied groups (DCDII vs. EPD, *p < 0.05). **(D)** eGFR in Maastricht class II DCD, Maastricht class III DCD, and EPD recipients (DCDII vs DCDIII, °*p* < 0.005; EPD vs. DCDII, *p < 0.001). **(E)** Kaplan-Meier curve of graft survival, by group (log rank test *p* = 0.408). **(F)** Kaplan-Meyer curve of patient survival, by group (log rank test *p* = 0.245).

Immediate recovery of renal function was noted predominantly in the EPD group (EPD 76.19%, DCD 29.4%, *p* < 0.0001), while the DGF rate was higher in the class II DCD group than in the EPD group (class II DCD: 76.47%, class III DCD: 38.89%, EPD 23.81%) (Figure 2A).

The need for dialysis was higher in class II than in class III DCD and EPD recipients (Figure 2B). Hospital length of stay was significantly higher in the class II DCD recipients than in EPD recipients (*p* < 0.05) (Figure 2C).

### Graft Function

During the follow-up period, the highest eGFR was observed in the EPD group, but it was not significantly different from that in the class III DCD group (Figure 2D). Interestingly, the eGFR was higher in class III than in class II DCD, but the difference among the two groups was significant only for the first month after transplant. In addition, eGFR was similar in EPDc and EPDn (Supplementary Figure S1).

### Graft and Patient Survival

Graft survival and patient survival were similar in the DCD and EPD groups, as shown by Kaplan-–Meier curves in Figures 2E,F. All deceased patients had functioning grafts.

Causes of death are described in Supplementary Table S1.

### Medical and Surgical Complications Post-Transplant

The rates of post-transplant complications causing graft loss did not differ among the DCD (11%) and EPD (4.5%) groups.

In the DCD group, three grafts were explanted because of renal vein thrombosis, severe hemorrhage secondary to a mycotic aneurysm, or severe life-threatening sepsis requiring immunosuppressive therapy suspension.

The surgical complication rate was higher in the DCD group than in the EPD group: lymphocele was observed in four DCD recipients (11.1%), urinary leakage in two DCD recipients (5.5%), and perirenal hematoma in two EPD (9%) and one DCD recipient (2.7%).

Kidney acute rejection (Banff 2a) occurred in only one patient belonging to the EPD group.

## Discussion

We report the renal transplantation results after 11 years of follow-up, by comparing the outcomes for EPD and DCD grafts. Our data showed excellent clinical outcomes in the recipients belonging to all groups. EPD was revealed to be a novel and promising category of donor, that has not been taken into consideration previously.

The EPD recipients achieved better outcomes than the DCD recipients. They showed better renal function, lower DGF rates, reduced dialysis need, shorter post-transplant hospital stays, and lower short- and long-term medical and surgical complication rates. However, the two groups of recipients showed no differences in PNF or graft and patient survival rates. Several factors, both immunological and non-immunological, are known to affect DGF occurrence and influence graft outcomes.

The emergence of new therapies as well as the advancements in mesenchymal stem cell and growth factor therapies and drug monitoring have improved the graft outcomes (12–17), but reduction of risk factors to prevent organ failure remains an important step.

Donor-related risk factors include age, body weight, cause of death, CIT, and WIT (9, 18), while recipient-related factors include the time spent on dialysis, obesity, diabetes, age, and race (18–22). However, WIT remains the most critical determinant of renal tissue injury, which is also related to DGF occurrence (9, 23–24). Despite the limitations of a retrospective study, the donors and recipients of the groups in this study had similar demographic and clinical characteristics; since they showed no differences in the average CIT, NRP, and HPM times. The only significant differences were noted in relation to WIT, which was longer in the DCD group than in the EPD group; notably, the maximum WIT was found in the class II DCD group, and the lowest value was observed in the EPDn group. Pure WIT was similar in class II DCD and EPDc groups; however, it was shorter in the class III DCD group and even shorter in the EPDn group. WIT is known to be an independent risk factor for DGF and acute kidney injury (23, 24). Our findings confirm the harmful influence of WIT on graft outcome. WIT is a hemodynamic impairment that implies a cessation of oxygen and nutrient delivery to the tissues and accumulation of metabolic waste products, which is followed by endothelial and epithelial necrosis, severe inflammation and immune cell activation, and a frequently maladaptive repair process, all of which lead to fibrosis. The pathogenesis of kidney fibrosis induced by ischemia remains an unresolved issue. The nature and the exact moment of the molecular switch between renal tubular repair and progression to atrophy/fibrosis as a response to injury is currently unknown (25). The successful outcomes of grafts from EPD support the hypothesis that early ECMO application could protect renal tissue from severe ischemic injury and predispose the tissue to switch to the correct repair mechanism. The main benefit derived from the immediate application of the ECMO device in the EPD group is the possibility of restoring stable blood circulation (i.e., a mean arterial pressure ranging from 50 to 60 mmHg, an SaO_2_ value ranging from 98 to 100%, good gas exchange, and a normothermic body temperature), which ensure good tissue perfusion (26–29).

In fact, the advantage derived from ECMO explains why eGFR was not different between the EPDc and EPDn subgroups, although pure and total WIT were significantly lower in the EPDn subgroup.

In contrast, in DCD, extracorporeal circulation is performed as a method of organ preservation only after the patient’s death declaration; therefore the long unstable circulatory period affects the performance of organs. Few reports have investigated the influence of total-body ECMO on donors arising from an unsuccessful extracorporeal life support treatment, and its advantages are unclear. In contrast, several clinical and animal studies have already proven the efficacy of NRP in reversing warm ischemic damage (30–33).

On the other hand, in our protocol, early application of ECMO was aimed at patient resuscitation and was not meant for organ procurement. Interestingly, total-body ECMO, which was applied to assist the circulatory and respiratory functions in DBD, reduced the ischemic damage caused by amines and improved organ quality, leading to a decrease in organ discard rates (33). This effect seems to depend on cellular energy restoration, as supported by studies in animal models (12, 13, 32). Moreover, some authors demonstrated that ECMO in cardiogenic shock was associated with lower levels of systemic inflammation (34). Thus, it could be argued that the comparison between DCD and EPD is improper because EPDn is more similar to DBD than to DCD.

Indeed, the EPDn recipients were subjected to a shorter WIT than the others, because death certification occurs by neurological criteria but these donors cannot be considered the same as DBD donors because they suffer from a refractory cardiac arrest or a cardiogenic shock; thus, the patients can remain on ECMO for hours, days, or weeks before the treatment is declared to be futile and unsuccessful. We would like to emphasize that EPD donors undergo a warm ischemia period during the asystolic phase as the class II DCD. Finally, ECMO provides artificial blood circulation through a roller pump, which cannot induce physiological cardiac systole and diastole as in DBD.

In summary, we have provided preliminary evidence showing that ECMO, applied before the patient’s death declaration, protects the kidney against ischemic injury, as demonstrated by the higher eGFR achieved with EPD grafts than with DCD grafts throughout the follow-up period. Furthermore, our results support the use of DCD despite the higher rate of DGF, but no study has yet reported the excellent long-term outcome of kidney transplants from DCD with a 20 min “no-touch period.” Patient and graft survival rates do not significantly differ in kidney transplants from DCD and EPD.

We are aware of the methodological limitations of the study, including the retrospective approach. The lack of controls selected according to a priori criteria precluded definitive conclusions. Nevertheless, we wish to highlight how EPD may be considered a new source of donors with excellent outcomes, at least for kidney transplants. We will test this hypothesis in a sound prospective investigation.

In the Maastricht class II DCD group, the cardiac arrest may occur out of - or in-hospital witnessed by standers. Resuscitation maneuvers are performed to save the patient’s life. WIT consists of a no-flow time (circulatory time elapsed from the cardiac arrest to the beginning of ACLS plus a 20 min no-touch period) and a low-flow time (up to a maximum of 120 min), during which a basic level of oxygenated blood circulation is restored by means of a cardiac compressor and mechanical ventilation.

Maastricht class III DCD typically includes an unpredictable agonal phase (maximum 2 h) following the WLST and a no-flow period while the EKG is recorded. Therefore, WIT consists of the time of the agonal phase plus the no-touch period. EPD is a type of donor resulting from an unsuccessful ECMO treatment after an irreversible cardiac arrest. In this setting, the patient’s death can be certified by either cardiac (EPDc) or neurological criteria (EPDn). The first choice requires recording a 20 min EKG (no-touchperiod). In contrast, if the patient’s death is certified by the neurological criteria the no-touch period can be avoided. After death determination, all types of donors undergo regional normothermic perfusion aimed at preserving the abdominal organs until harvesting. The removed organ is subsequently stored in the mechanical pulsatile perfusion machine until transplantation.

## Data Availability

The raw data supporting the conclusion of this article will be made available by the authors, without undue reservation.

## Supplementary Material

Supplementary Figure 1 eGFR in EPDn and EPDc recipients.

## Abbreviations

ACLS, advanced cardiovascular life support; BMI, body mass index; CIT, cold ischemia time; CKD-EPI, chronic kidney disease epidemiology collaboration; CPR, cardiopulmonary resuscitation; CS, cold storage; DBD, donation after brain death; DCD, donation after circulatory death; DGF, delayed graft function; ECMO, extracorporeal membrane oxygenation; EEG, Electroencephalography; eGFR, estimated glomerular filtration rate; EKG, electrocardiogram; EPD, ECMO prior to death donation/donor; EPDc, patient’s death certified by cardiac criteria; EPDn, patient’s death is certified by neurological criteria; ERSD, end stage renal disease; HLA, human leukocyte antigen; HMP, hypothermic perfusion machine; MP, machine perfusion; NRP, normothermic regional perfusion; PNF, primary non function; RR, renal resistances; WIT, warm ischemia time.
