# Supplementary material for: Kidney Transplants From Donors on Extracorporeal Membrane Oxygenation Prior to Death Are Associated With Better Long-Term Renal Function Compared to Donors After Circulatory Death
Source: Transpl Int. 2022 Feb 8;35:10179. doi: 10.3389/ti.2021.10179 (PMC8862176; doi:10.3389/ti.2021.10179)
Supplement: Supplementary file 1 [file Table1.docx]

Supplementary Table 1: characteristics of not-survived patients.

| **ID RECIPIENTS** | **DAYS OF SURVIVAL POST TX** | **CAUSE OF DEATH** | **DONOR CLASS** | **SEX AND DEATH AGE** |
| --- | --- | --- | --- | --- |
| DCD1 | 3076 | PULMONARY ASBESTOSIS | II | M 75 |
| DCD2 | 90 | STROKE | II | F 59 |
| DCD3 | 2750 | CHOLANGICARCINOMA | II | M 70 |
| DCD4 | 342 | CMV PNEUMONIA | III | M 71 |
| EPD1 | 2517 | HEART SURGERY COMPLICATION | EPD | M 42 |
| EPD2 | 2415 | BREAST CANCER | EPD | F 49 |
| EPD3 | 118 | HEART ATTACK | EPD | M59 |
| EPD4 | 83 | STROKE | EPD | M64 |
