# Supplementary figures and images for: Kidney Transplants From Donors on Extracorporeal Membrane Oxygenation Prior to Death Are Associated With Better Long-Term Renal Function Compared to Donors After Circulatory Death
Source: Transpl Int. 2022 Feb 8;35:10179. doi: 10.3389/ti.2021.10179 (PMC8862176; doi:10.3389/ti.2021.10179)

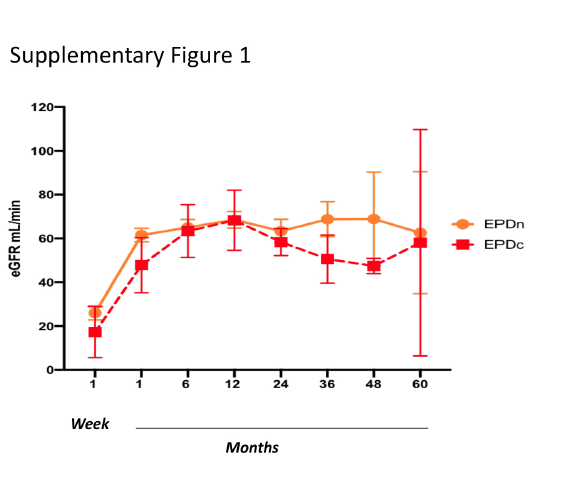

Supplement: Supplementary file 2 [file DataSheet1.docx]
